# Drug Proving

**Published:** 1881-07

**Authors:** Ad. Lippe

**Affiliations:** Philadelphia


### DRUG PROVING.

Ad. Lippe, M. D., Philadelphia.

Our knowledge of the curative virtues of drugs depends on our knowledge of their sick-making properties; this latter can be obtained only by proving drugs on the healthy. Hence, it is evident, that the *true* healer will never treat the sick with unproved drugs.

The object of this paper is to offer some suggestions for the proving of drugs, as it is obvious that the progress of the Healing Art depends solely upon an increased and thorough knowledge of the sick-making powers of drugs. Hahnemann has given us, in *The Organon*, full reasons for the necessity of proving drugs, as well as directions how to do it. It would seem superfluous to reiterate the arguments he gave us almost fifty years ago, were it not self-evident that numbers of his professed followers are not conversant with the teachings to be found in said *Organon*.

We now address ourselves principally to just such men; they are imitating a vain attempt, made more than half a century ago by a Dr. Lux, to introduce into our therapeutics the unproved products of disease, which he claimed would, when potentized, cure the same disease. Hahnemann alludes to this "departure" in a foot-note to paragraph 56 of his *Organon.* If all persons coming under the influences of a miasm were affected precisely alike, then only would it be rational to apply the potentized product of this miasm for the cure of it; but as it is well known that different persons are very differently affected by each miasmatic and contagious disease, it is obvious that a generalization, as proposed again nowadays, cannot be accepted. Homœopathy individualizes, while the common school of medicine generalizes. All medical men who indulge in the belief that pathology has become an exact science; that the modern theories as to diseases are true, or any truer than the former ever-changing hypotheses; that we, as homœpathists, should take these modern hypotheses and incorporate them into our Healing Art, and, through them, find specific remedies for specific diseases; all those who go further astray, and indulge in the fallacious belief that the product of a disease when potentized—highly potentized—will cure, permanently cure, the same disease in others, these medical men will find that they have been running after a phantom. This phantom-hunt consists in seeking a fixed form of disease, pathologically labeled, and presented to innocent students of the Healing Art, in works on pathology, or on diagnosis on the part of the common school; and by such works as the "Pharmacodynamics," on the part of homœopathists. These phantoms make the unfortunate seeker for wisdom believe, that he has found finally a specific remedy for a specific disease. Sooner or later the reality will stare this unfortunate and deceived young Æsculapius in the face, that his "specifics for specific diseases" are an illusion and a snare, notwithstanding that the teacher who allured him into this fallacious belief may have stood high in an Allopathic University, or stood high on a Potentizer proclaiming such "specifics." The deluded one may then read earnestly *The Organon* of Samuel Hahnemann, and make the experiment as he teaches him to make it. Then he will abandon the phantom, and become a true Healer. As this paper may reach just such unfortunate, but honestly intentioned men, who are in want of the light, which they can obtain only by reading *The Organon*, we can but ask them to see what Hahnemann did say on this subject, and become interested in the study of the most philosophical and logical medical work ever written by inspired man on Healing Art—*The Organon* of Samuel Hahnemann.

Our first question is—*Who should prove drugs?*

Every one in a tolerable state of health, able to observe on himself any changes that may take place, different from his ordinary feelings and sensations, is able to prove a medicine. The more diversified the constitution, disposition, age and sex of the provers, the better will be the provings.

To be most fully prepared for the task he is undertaking, the prover should note down his daily state of health for a week before he begins his provings. He will then find it much easier to describe such sensations and feelings as deviate from his usual normal condition. The art of observation is one of the most important faculties to be learned by the Healer. Nothing will aid him more in the acquisition of this art than self-examination. Proving of drugs will be more fruitful in developing this self-observation than anything else. Once acquired, it will make the art of observation upon others a comparatively easy task. Skill in proving, leads to skill in examining the sick; and having, as a prover, carefully observed all the minutest symptoms caused by the drug, one will almost involuntarily compare these new symptoms with those produced by other (already proved) drugs, and obtain, by such comparisons, an insight into our materia medica, which he could not possibly acquire in any other way.

### THE DRUG TO BE PROVED.

The first object is to procure the drug or other matter to be proved in its purity; then to make a full statement as to how and where it was obtained and how it was prepared. The preparation of chemical substances was always given in detail by Hahnemann, so as to insure the reproduction of precisely the same chemical substance in the future. Plants should be collected by the prover, if possible, at the right season and where they grow on their original soil; for instance, a flower taken from the Cactus grandiflorus growing in a hot-house will not make a good preparation, either for provings or as a curative agent. This preparation should be made, as it was made, on the spot where the Cactus grows wild, and at the right time and season, when the flower opens at night and fills the atmosphere with its fragrance.

If the drug be taken from the animal kingdom, the animal should, if possible, be preserved and subsequent supplies should come from the same species, and under similar circumstances. The few drops of poison taken from the Trigonocephalus Lachesis by Dr.

Hering in Surinam, over fifty years ago, has sufficed so far to supply all the demand for Lachesis. What is more, the identical snake from which the poison was taken is still preserved in the Academy of Natural Sciences of Philadelphia. Preparations taken from the same species of snake, while confined in cages in menageries or any public institutions, cannot reasonably be expected to have the same medicinal power as those from the wild snake brought alive to Dr. Hering by the Indians in the country where it was caught.

### DOSES.

We know that one contact with an infectious disease, one inhalation of malarious air, one sudden mental emotion, will cause a succession of phenomena and symptoms, which finally end either in a full recovery, by what is termed the crisis or throwing off of the diseased condition of the organism; or else, if the organism be in too feeble a condition to resist the influences, or if the effort of Nature to bring about this crisis have been interfered with by violent means, (*i. e.*, energetic treatment,) the system succumbs to the overpowering influences, and death is the consequence.

This observation of the natural causes of natural diseases must serve us as a guide in ascertaining the sick-making properties of drugs. If we wish to ascertain the artificially diseased condition drugs produce upon the healthy, we make our experiment by taking one dose of the drug; and as we do not expect an immediate effect from a contact with an infectious disease, experience teaching us that it requires days, hardly ever less than three days, before the effects of such a contact become perceptibly developed, so we cannot reasonably expect an immediate perceptible development of the sick-making effects of the one dose of the medicine to be proved. If there is no effect perceptible after, say five days, we will have to proceed just as we do when we administer medicines for the cure of the sick; finding ourselves not susceptible to the drug to be proven, we must take either a lower or higher preparation; and when no effects follow this, we may take the potentized drug in a watery solution until an effect is perceptible. When the question arises what preparation of the drug we should take in that *one* first dose, we may as well consult Hahnemann, who tells us, in paragraph 128 of his *Organon*, that substances, if proved in the crude state, by no means show the richness and fullness of their sick-making powers; that the dormant powers of the drug are developed by potentization; and that we obtain a better knowledge of the properties of drugs if we take a few pellets of the 30th potency. Fifty years ago the 30th potency was the highest potency known, since then innumerable experiments, both on the healthy and the sick, have fully established the fact that a greater degree of sick-making power is developed by much higher potentizations. When Hahnemann advised a few pellets of the 30th potency as a proper dose for testing the drug, knowing that its medicinal powers are developed by potentization, his followers tried the experiment, and ascertained that the highest known potencies are endowed with a proportionately higher medicinal property than the crude substances or lower preparations possess. All depends upon the only reliable test, experiment; whoever will make this experiment honestly, will find that a single dose of the highest potency will cause a succession of symptoms much more distinctly marked, much more characteristic than any other preparations before used, even in the single dose or in repeated doses. We have, for instance, this day, no other provings of Theridion than those made by the 30th potency, we have provings of Lachnanthes made by the highest potency then known (76 m.) and the symptoms obtained in this manner have been confirmed by clinical experiment.

### REGIMEN DURING THE PROVING.

The prover will do best to continue his usual diet and habits in general, as a deviation from them would necessarily cause some changes in his condition, and these might erroneously be attributed to the effects of the drug he proves. At the same time, he should, for this same reason, avoid all possible mental excitement and, above all, any exposures to the changes of the weather or to cold. Such exposures, during the development of the sick-making properties of a drug, might, as we know it did in several deplorable instances, fix upon the prover ailments for life. We know that a person suffering from an acute disease has to be very careful not to expose himself to influences of mental disturbance or the weather, which in his ordinary state of health, would affect him only temporarily; but which, during an acute illness might, and often does, leave their marks, disturbing his health during the rest of his life.

### THE DAY-BOOK.

The prover would do well to give first a description of himself—age, sex, temperament, former ailments or diseases, habits and the influence which changes in the weather have on him. Next, a full description of the substance or drug proved, how and where it was obtained and how it was prepared. Next mention the dose and the time of the day. This self-examination should be as carefully conducted as the examination of a sick person. A daily journal should be kept, in which nothing is omitted; some symptoms, or groups of symptoms, may often reappear, they should be very distinctly related again, as these frequently recurring disturbances, however long they may continue, often denote the most characteristic symptoms of the substance or drug proved. And, as in the examination of the sick, so in proving, the experimenter should describe very minutely under what circumstances certain symptoms appear. Also state whether food, changes in the weather, exercise or rest in certain position, cause new, or aggravate, or ameliorate old symptoms.

Finally, let us remember that the proving of drugs of all kinds and by many persons, will not only increase our ability to cure the sick, but will also forever settle many, as yet, disputed points, such as the possibility of finding a drug which can produce symptoms forming the exact similar to a known pathological condition—a disease. Proving will settle forever the disturbing posological question; provings, and their practical utilization, will confirm the infallibility of the only Law of cure—*Similia similibus curantur.*

